# Association between serum insulin-like growth factor 1 and locomotive syndrome in community-dwelling older people

**DOI:** 10.1186/s12891-022-05738-3

**Published:** 2022-08-11

**Authors:** Misa Nakamura, Masakazu Imaoka, Hidetoshi Nakao, Mitsumasa Hida, Fumie Tazaki, Ryota Imai, Hiroshi Hashizume

**Affiliations:** 1 Cognitive Reserve Research Center, Osaka Kawasaki Rehabilitation University, Mizuma, 1558, Kaizuka City, Osaka 597-0104 Japan; 2 Department of Rehabilitation, Osaka Kawasaki Rehabilitation University, Mizuma, 1558, Kaizuka City, Osaka 597-0104 Japan; 3 Department of Orthopaedic Surgery, Wakayama Medical University, Kimiidera 811-1, Wakayama City, Wakayama 641-8510 Japan; 4 School of Health and Nursing Science, Wakayama Medical University, Mikazura 580, Wakayama City, Wakayama 640-0011 Japan

**Keywords:** IGF-1, Locomotive syndrome, Community-dwelling, Older people

## Abstract

**Background:**

Locomotive syndrome (LS) is a condition in which mobility decreases, and it is known as a risk factor for elderly persons needing care in connection with sarcopenia and frailty. Prevention or delay of the onset of these diseases is important for preventing the need for care, and identification of biomarkers as indicators for appropriate intervention is useful. The present study aimed to clarify whether the serum insulin-like growth factor 1 (IGF-1) level, which has been reported to be related to sarcopenia and frailty, is related to LS.

**Methods:**

The study participants were 133 elderly people living in a rural area in Japan. LS was assessed using Locomo-25, which is a self-administered questionnaire, and LS was defined as a Locomo-25 score ≥ 7 points. Serum IGF-1 and albumin levels were measured. A self-completed medical history questionnaire was used.

**Results:**

On multiple linear regression analysis, age, IGF-1, osteoporosis, and osteoarthritis were significantly associated with the Locomo-25 score. The receiver-operating characteristic curve analysis of the IGF-1 level showed a threshold value of 82.0 ng/mL for discriminating non-LS and LS. The logistic regression analysis adjusted for osteoporosis, osteoarthritis, and the propensity score estimated from sex, age, and BMI showed that the odds ratio (OR) of the IGF-1 level for LS was 1.019 (95% confidence interval [CI], 1.002–1.039; *p* = 0.027), and the OR of IGF-1 ≤ 82 ng/mL for LS was 2.275 (95% CI 0.993–5.324; *p* = 0.052).

**Conclusions:**

The present findings suggest that osteoporosis and osteoarthritis were associated with early LS, and a decrease of the serum IGF-1 level was a significant independent factor for early LS.

## Background

Of the causes for needing long-term care in Japan, weakness due to old age is third, fracture/fall is fourth, and joint disease is fifth [1]. The concept of frailty has been viewed as a pre-stage of debility due to old age, which is the third leading cause of needing long-term care [2]. According to the definition of frailty, muscle weakness is the main pathological condition of the physical element of frailty.

The Japanese Society of Orthopedic Surgery has proposed the concept referred to as locomotive syndrome (LS), which is a decline in locomotive function associated with a high risk of requiring long-term care as it progresses [3]. We have previously reported on the relationships between LS and motor function, body composition, depression, cognitive function, and oral function in elderly persons living in the community [4–8].

On the other hand, in recent years, age-related muscle loss, sarcopenia, has been attracting attention as a cause of muscle weakness. Sarcopenia is a concept that mainly involves a decrease in muscle mass and also includes a decrease in function, such as decreases in grip strength and walking speed [9], but it can be said to be a causative disease of LS. Therefore, one can see that frailty, sarcopenia, and LS are not completely independent concepts of disease, but are related to each other. Prevention or delay of the onset of these diseases is important to prevent the need for care [10, 11], and identification of biomarkers as indicators of appropriate intervention is beneficial.

Insulin-like growth factor 1 (IGF-1) is produced in many tissues, including osteocytes and muscle cells, and it functions as a myokine that regulates myogenesis and muscle hypertrophy [12]. On the other hand, IGF-1 contributes to bone formation [13]. Hamrick et al. showed that IGF-1 is present in vivo at the boundary between muscle and bone, and it is abundant in muscle tissue and secreted from muscle tubes cultured in vitro [14]. Therefore, muscle hypertrophy and bone formation are thought to be synchronized by the IGF-1 paracrine signal [15]. Previous reports have shown that lower IGF-1 blood levels are associated with frailty and sarcopenia in older persons, as well as cardiovascular disease, diabetes mellitus, cancer, and cognitive impairment [16–23].

The present study aimed to clarify whether the serum IGF-1 level, which has been reported to be related to sarcopenia and frailty, is related to the LS. This is expected to be useful for the detection of LS progression.

## Methods

### Participants

This was a cross-sectional, population-based study conducted in Kaizuka-City, Osaka Prefecture, Japan, between 2019 and 2020. The inclusion criteria were as follows: age ≥ 65 years; living independently at home; and not having a cardiac pacemaker. Study participants were recruited from a pool of individuals who had participated in a local government-supported check-up for health in Kaizuka-City. In total, 133 participants were analyzed, after excluding two who had incomplete data. This study was approved by the Ethics Committee of Osaka Kawasaki Rehabilitation University (Reference No. OKRU30-A016) and performed in accordance with the Declaration of Helsinki. Written, informed consent was obtained from all participants before the study began.

### Measurements of body composition

Body composition parameters were measured using a bioelectrical impedance analysis (BIA) device (InBody 270; InBody, Tokyo, Japan) at 20 and 1000 kHz while the participants were wearing normal indoor clothing without socks or shoes [24].

All participants were instructed to grasp the handles of the BIA device and stand on electrodes contacting the bottoms of their feet. The body mass index (BMI) was calculated as weight in kilograms divided by height in meters squared (kg/m^2^). The skeletal muscle mass index (SMI) was calculated as muscle mass in kilograms divided by height in meters squared (kg/m^2^). Calcaneal bone mineral density (BMD) was evaluated by quantitative ultrasound (i.e., the speed of sound [SOS] of the calcaneus) and expressed as the percent of the young adult mean of the SOS (%YAM) using an ultrasound bone densitometer (AOS-100SA; Hitachi, Tokyo, Japan). All participants placed their right heel on the quantitative ultrasound device while seated, and it is expressed as the percent of the young adult mean of the SOS (%YAM).

### Assessment of LS status

LS status was evaluated using the Locomo-25 score, which is a self-administered questionnaire composed of four questions about pain, 16 questions about activities of daily living, three questions about social function, and two questions about mental health status during the last month [25]. All 25 items are scored from 0 (no impairment) to four (severe impairment), with the total score ranging from 0 to 100. A Locomo-25 total score of ≥7 points is the cut-off point for the diagnosis of LS, with a Locomo-25 total score of 7–15 points classified as LS stage 1, 16–23 points as stage 2, and ≥ 24 points as stage 3 [3].

### Serum biochemical measurements

All participants fasted 2 h before blood collection, and blood samples were drawn between 10:00 and 15:00. IGF-1 and albumin levels were measured. Albumin was used as a nutritional marker. Blood analyses were performed at a laboratory within 24 h of collection (Japan Clinical Laboratories, Inc., Kyoto, Japan). Total IGF-1 and albumin levels were measured by radioimmunoassay and nephelometry, respectively.

### Medical history questionnaire

A self-completed medical history questionnaire regarding hypertension, diabetes mellitus, hyperlipidemia, osteoporosis, and osteoarthritis was administered.

### Statistical analysis

On univariable linear regression analysis, sex, age, serum IGF-1 level, serum albumin level, BMI, SMI, BMD, and medical history were used as dependent variables, and the Locomo-25 score was the independent variable. On multiple linear regression analysis, sex, age, serum IGF-1, BMI，osteoporosis, and osteoarthritis were used as dependent variables, and the Locomo-25 score was the independent variable. The IGF-1 threshold values for discriminating non-LS and LS were evaluated by receiver-operating characteristic curve (ROC) analysis. The odds ratios (ORs) of IGF-1 levels or IGF-1 status threshold values for LS were calculated using multiple logistic regression analyses. IGF-1 levels or IGF-1 threshold values were used as dependent variables adjusted by the presence or absence of osteoporosis and osteoarthritis, and the propensity score estimated from sex, age, and BMI. The LS was the independent variable. Statistical analysis was conducted using JMP 11 (SAS Institute, Cary, NC). All statistical tests were two-tailed, and a significance level of 0.05 was used.

## Results

### Characteristics of the study participants

Age, serum IGF-1 level, albumin level, BMI, SMI, BMD, medical history, Locomo-25 score, and LS stage by sex are shown in Table 1.

**Table 1:** Participants’ characteristics by sex

	Male	Female
Participants	24 (18.05%)	109 (81.95%)
Age, y	74.67 (6.26)	74.68 (5.06)
IGF-1, ng/mL	94.38 (26.27)	89.20 (25.88)
Albumin, g/dL	4.28 (0.25)	4.30 (0.25)
BMI, kg/m^2^	23.88 (3.19)	22.77 (3.20)
SMI, kg/m^2^	7.28 (0.89)	5.64 (0.64)
BMD, %YAM	93.54 (10.81)	84.92 (10.45)
Hypertension	11 (45.83%)	50 (45.87%)
Diabetes mellitus	3 (12.50%)	7 (7.52%)
Hyperlipidemia	7 (29.17%)	33 (30.28%)
Osteoporosis	0 (0%)	38 (35.19%)
Osteoarthritis	1 (4.17%)	21 (19.27%)
Fracture after age 60 y	2 (8.33%)	19 (17.59%)
Locomo-25 score, points	6.38 (6.64)	8.38 (10.23)
LS	8 (33.33%)	39 (35.78%)
LS stage 1	4 (16.67%)	21 (19.27%)
LS stage 2	4 (16.67%)	9 (8.26%)
LS stage 3	0 (0%)	9 (8.26%)

Values are presented as means (standard deviation) or prevalence (%)

*IGF-1* insulin-like growth factor 1, *BMI* body mass index, *SMI* skeletal muscle mass index, *BMD* bone mineral density, *%YAM* % of young adult mean of the speed of sound of the calcaneus, *LS* locomotive syndrome, *LS stage 1* Locomo-25 total score of 7–15 points, *LS stage 2* 16–23 points, *LS stage 3* 24 points or more

### Coefficients for factors associated with the Locomo-25 score on univariate linear regression analysis

The univariate linear regression analysis results to identify variables associated with the Locomo-25 score are shown in Table 2. Age (β = 0.323, *p* < 0.001), IGF-1 (β = − 0.219, *p* = 0.011), osteoporosis (β = 0.273, *p* = 0.002), and osteoarthritis (β = 0.339, *p* < 0.001) were significantly associated with the Locomo-25 score (Table 2).

**Table 2:** Results of linear regression analysis for each factor and its association with the Locomo-25 score

	β	95% CI	*P*
Sex	0.080	−2.323, 6.325	0.362
Age	0.323	0.293, 0.895	< 0.001
IGF-1	−0.219	− 0.145, − 0.019	0.011
Albumin	− 0.065	−9.185, 4.148	0.456
BMI	0.094	−0.234, 0.802	0.280
SMI	−0.076	−2.558, 0.997	0.387
BMD	−0.124	−0.231, 0.041	0.154
Hypertension	0.094	−1.514, 5.153	0.282
Diabetes mellitus	0.008	−6.018, 6.634	0.923
Hyperlipidemia	0.098	−1.568, 5.673	0.264
Osteoporosis	0.273	2.285, 9.383	0.002
Osteoarthritis	0.339	4.581, 13.029	< 0.001
Fracture after age 60 y	0.034	−3.685, 5.459	0.702

*IGF-1* insulin-like growth factor 1, *BMI* body mass index, *SMI* skeletal muscle mass index, *BMD* bone mineral density, *%YAM* % of young adult mean of the speed of sound of the calcaneus, *CI* confidence interval

### Coefficients for factors associated with the Locomo-25 score on multiple linear regression analysis

Multiple linear regression analysis was performed with the factors, and age (β = 0.266, *p* = 0.001), IGF-1 (β = − 0.164, *p* = 0.033), osteoporosis (β = − 0.273, *p* = 0.001), and osteoarthritis (β = − 0.302, *p* < 0.001) were found to be significantly associated with the Locomo-25 score. The variance inflation fractions (VIFs) of all variables were less than two, meaning no multi-collinearity (Table 3). The results of univariate linear regression analysis showed no relationship between the Locomo-25 score and BMI, but since our previous study showed a relationship between LS and BMI [5], BMI was added to this analysis.

**Table 3:** Results of multiple linear regression analysis for factors associated with the Locomo-25 score

	Coefficient (β)	95% CI	*P*	VIF
Sex	0.044	−1.108, 2.510	0.579	1.132
Age	0.266	0.216, 0.763	0.001	1.025
IGF-1	−0.164	−0.117, −0.005	0.033	1.046
BMI	0.131	−0.081, 0.869	0.103	1.156
Osteoporosis	−0.273	−4.636, −1.203	0.001	1.199
Osteoarthritis	−0.302	−5.885, −1.949	< 0.001	1.066

*β* standardized partial regression coefficient, *IGF-1* insulin-like growth factor 1, *CI* confidence interval, *VIF* variance inflation factor

### Threshold values of IGF-1 for LS status

ROC analysis of the IGF-1 level showed a threshold value of 82.0 ng/mL for discriminating non-LS and LS (area under the curve = 0.618, sensitivity = 55.32%, specificity = 66.28%, *p* = 0.014) (Table 4).

**Table 4:** Threshold value of the IGF-1 concentration for LS

Threshold values of IGF-1 (ng/mL)	AUC	Sensitivity (%)	Specificity (%)	*p*
82.00	0.618	55.32	66.28	0.014

ROC (receiver-operating characteristic curve) analysis was performed

*IGF-1* insulin-like growth factor 1, *LS* locomotive syndrome, *AUC* area under the curve

### Odds ratios for LS according to IGF-1

The logistic regression analysis showed that participants with IGF-1 had an OR of 1.019 (95% confidence interval [CI] 1.002–1.039; *p* = 0.027) for LS adjusted by osteoporosis, osteoarthritis, and the propensity score estimated from sex, age, and BMI. Furthermore, logistic regression analysis showed that participants with IGF-1 ≤ 82.00 ng/mL had an OR of 2.275 (95% CI 0.993–5.324; *p* = 0.052) for LS adjusted by osteoporosis, osteoarthritis, and the propensity score estimated from sex, age, and BMI (Table 5).

**Table 5:** Odds ratios for LS by the IGF-1 value

	Odds ratio	95% CI	*p*
Model 1			
IGF-1(− 1 ng/ml)	1.019	1.002, 1.039	0.027
Model 2			
IGF-1 (< 82 ng/mL)	2.275	0.993, 5.324	0.052

The multiple logistic regression analysis was adjusted by presence or absence of osteoporosis and osteoarthritis, and propensity score estimated from sex, age, and BMI

The explanatory variable is the numerical value of IGF-1 in Model 1, and the nominal variable with IGF-1 < 82 in Model 2

*CI* confidence interval, *IGF-1* insulin-like growth factor 1, *LS* locomotive syndrome

## Discussion

This cross-sectional study of community-dwelling elderly persons showed an association between the IGF-1 level and LS. In other words, elderly people with low blood IGF-1 levels had a high degree of LS. The relationship between the serum IGF-1 level and the Locomo-25 score remained significant after adjusting for several covariates, including locomotor disorders. The participants with decreased IGF-1 level had a significant OR for LS adjusted by osteoporosis, osteoarthritis, and propensity score estimated from sex, age, and BMI. ROC analysis showed that the cut-off value to distinguish between non-LS and LS was 82 ng/mL. The logistic regression analysis adjusted for osteoporosis, osteoarthritis, and the propensity score estimated from sex, age, and BMI showed that IGF-1 ≤ 82 ng/mL was not significant for LS (*p* = 0.052), but the OR was 2.275 and the 95% CI was 0.993–5.324, suggesting that this cutoff value is useful for distinguishing between non-LS and LS.

In the current LS stage diagnosis, LS stage 1 is a state in which movement function has begun to decline, stage 2 is a state in which the movement function is declining progressively, and stage 3 is a state in which the movement function has declined even further and is hindering social participation [3]. The present study suggested that the serum IGF-1 level could be a biomarker to predict LS stage 1.

Ito et al. reported that the ratio of nonmercaptalbumin (HNA) to albumin (HMA), a marker of metabolic stress, can be a biomarker for LS in elderly subjects (≥ 65 years) [26]. The results for the relationship between a high HNA/HMA ratio and a high LS stage were thought to be related to the increase in oxidative stress and the exacerbation of LS. In the present study, HMA was measured, but HNA was not measured, so the above relationship could not be analyzed.

Yoshihara et al. reported that high levels of glycated hemoglobin (HbA1c) and low levels of albumin in middle-aged and elderly people (40–85 years) were associated with the results of the start-up test and the 2-step test as LS diagnostic, but HbA1c and albumin levels had no relationships with LS using Locomo-25. Furthermore, no relationship between IGF-1 levels and LS based on the overall judgment of these three tests was also reported [27]. These results and the present results suggest that the difference in the method of determining LS reflects different physiological conditions.

It has been reported that there is a strong relationship between LS and walking speed [4, 28–30]. On the other hand, relationships between low IGF-1 levels and decreased knee extensor muscle strength and walking speed have been reported [31], and high IGF-1 levels show higher physical functions including walking speed in obese elderly people [32]. These previous reports strongly support the present results.

Growth hormone (GH) and its mediator, IGF-1, regulate many aspects of somatic cell growth, metabolism, and aging. GH/IGF action peaks during pubertal growth and regulates skeletal acquisition through stimulation of extracellular matrix production and increased BMD [33]. On the other hand, the GH/IGF axis is an important determinant of muscle mass and function in muscle [34]. Bones and muscles have been identified as secretory endocrine organs, and their interactions can affect their respective functions. In rodents, IGF-1 and IGF-2 are localized in muscle fibers along the muscle-bone interface of the mouse forefoot, and receptors for these growth factors are fleshy along the diaphysis of the long bone [35].

Osteoporosis, osteoarthritis [36], and spinal canal stenosis [37] are the main causative diseases of LS, and all of them involve musculoskeletal disorders. In the present study, spinal canal stenosis was not investigated, but relationships were found between LS and both osteoporosis and osteoarthritis. On the other hand, it has been reported that there is a relationship between the IGF-1 level and BMD [38], but it is unclear whether low levels of IGF-1 cause osteoporosis. IGF-1 is also involved in cartilage tissue, and IGF-1 promotes chondrocyte proliferation, enhances matrix production, and inhibits chondrocyte apoptosis. Therefore, IGF-1 is expected to be effective as a therapeutic agent for osteoarthritis [39].

Research is being conducted on creating profiling to increase the self-efficacy of exercise and encourage exercise [40]. With such an approach, it is possible that persons with LS may reduce their risk of acting through the IGF pathway by improving physical activity to the extent possible.

The present results suggest that IGF-1 can be a marker for LS. This result is similar to the decrease in serum levels of IGF-1 seen in frailty and sarcopenia [16–25]. According to the survey by Yoshimura et al., the number of people with frailty is estimated to be 2.2 million, the number of people with sarcopenia is estimated to be 3.7 million, and the number of people with LS is estimated to be 60 million, showing that the prevalence of LS is overwhelmingly higher than that of frailty and sarcopenia [41–43]. A study of the merger of frailty and sarcopenia with LS stage 1 and 2 showed that almost all people with stage 1 have frailty and sarcopenia [42]. In other words, the present results strongly support these findings.

Since exercise promotes the production of IGF-1 [44, 45], it is thought that a decrease in exercise and activity may also be a cause of the IGF-1 decrease in people with LS due to a disorder of the locomotive organs.

Studies in humans and animals suggest that increased serum IGF-1 levels may improve cognitive function [46, 47]. We previously reported a relationship between LS and cognitive function [7]. Motoric cognitive risk syndrome [48] and physio-cognitive decline syndrome [49] have been reported as conditions in which motor function decline and cognitive decline are observed with aging. It is possible that IGF-1 is involved in these syndromes.

### Limitations and future research

There are some limitations to this study. First, the number of subjects was small. The IGF threshold derived in this study is uncertain due to the small number of subjects. In the future, it will be necessary to increase the number of subjects for analysis. In addition, a longitudinal study is needed to observe the relationship between changes in IGF-1 concentrations and the development of LS. In addition to Locomo-25, there are currently a start-up test and a 2-step test for LS assessment, and in the future, it will be necessary to classify LS based on these three evaluations. This study clarified the relationship between LS and IGF-1, but the results of linear regression analysis showed that the β value of IGF-1 was lower than the β value of osteoporosis and OA, which means that it can be said that the relationship with IGF-1 is not as strong as that of motor organ disease. In the future, it will be necessary to investigate the process leading up to the onset of these locomotor disorders and how changes in IGF-1 are involved in LS.

## Conclusions

The present findings suggest that osteoporosis and osteoarthritis were associated with early LS, and a decrease of the serum IGF-1 level was also an independent factor related to early LS. Further study is needed to determine the threshold value for IGF to predict LS. Nevertheless, this is the first report to clarify the relationship between IGF-1 and LS.

## Data Availability

The datasets generated and/or analyzed during the current study are not publicly available due to limitations of ethical approval involving the participants’ data and anonymity, but they are available from the corresponding author on reasonable request.

## Abbreviations

IGF-1: Insulin-like growth factor-1
BMI: Body mass index
SMI: Skeletal muscle mass index
BMD: Bone mineral density
%YAM: % of young adult mean of the speed of sound of the calcaneus
LS: Locomotive syndrome
β: Standardized partial regression coefficient
CI: Confidence interval
VIF: Variance inflation factor
ROC: Receiver-operating characteristic curve
AUC: Area under the curve
OR: Odds ratio
